# Overcoming Barriers in Neurosurgical Education: Introducing a Simulator for Insular Glioma Resection with Fluorescence Imaging (SIGMA)

**DOI:** 10.3390/jcm14072479

**Published:** 2025-04-04

**Authors:** Sifian Al-Hamid, Vanessa Magdalena Swiatek, Julius Reiser, Firat Taskaya, Amir Amini, Klaus-Peter Stein, Ali Rashidi, I. Erol Sandalcioglu, Belal Neyazi

**Affiliations:** Department of Neurosurgery, Otto-von-Guericke University, 39120 Magdeburg, Germany; sifian.al-hamid@st.ovgu.de (S.A.-H.); vanessa.swiatek@med.ovgu.de (V.M.S.); julius.reiser@st.ovgu.de (J.R.); firat.taskaya@st.ovgu.de (F.T.); amir.amini@med.ovgu.de (A.A.); klaus-peter.stein@med.ovgu.de (K.-P.S.); ali.rashidi@med.ovgu.de (A.R.); erol.sandalcioglu@med.ovgu.de (I.E.S.)

**Keywords:** insular glioma, surgical simulation, surgical education, fluorescence imaging

## Abstract

**Background and Objectives:** Realistic surgical simulation models are essential for neurosurgical training, particularly in glioma resection. We developed a patient-specific simulation model designed for fluorescence-guided glioma resection, providing an anatomically accurate and reusable platform for surgical education. While insular gliomas were used as an example, the model can be adapted to simulate gliomas in other brain regions, making it a versatile training tool. **Methods:** Using open-source 3D software, we created a digitally reconstructed skull, brain, and cerebral vessels, including a fluorescent insular glioma. The model was produced through additive manufacturing and designed with input from neurosurgeons to ensure a realistic and reusable representation of the Sylvian fissure and bone structures. The simulator’s educational effectiveness and usability were evaluated by two senior physicians, four assistant physicians, and six medical students using actual microsurgical instruments. Assessments were based on subjective and objective criteria. **Results:** Subjective evaluations, using a 5-point Likert scale, showed high face and content validity. Objective measures demonstrated strong construct validity, accurately reflecting the participant’s skills. Medical students and resident neurosurgeons showed marked improvement in their learning curve over three attempts, with progressive improvement in performance. **Conclusions:** This simulation model addresses advanced neurosurgical training needs by providing a highly realistic, cost- effective, and adaptable platform for fluorescence-guided glioma resection. Its effectiveness in enhancing surgical skills suggests significant potential for broader integration into neurosurgical training programs. Further studies are warranted to explore its applications in different glioma localizations and training settings.

## 1. Introduction

Glioma surgeries pose significant challenges in neurosurgery, particularly when resecting both low-grade and high-grade gliomas. The complexity arises from their variable locations and proximity to critical vascular and cortical structures. Insular gliomas, for example, are among the most technically demanding due to their deep-seated position within the Sylvian fissure, surrounded by the frontal and lateral operculum [1,2]. Resection can be performed using either a transsylvian or transcortical approach, each with specific advantages and limitations, depending on biological, clinical, and anatomical factors [2,3]. Fluorescence-guided surgery, particularly with 5-aminolevulinic acid (5-ALA), has been shown to improve the extent of resection and facilitate the identification of tumor margins, making it a valuable tool for glioma surgery in various brain regions [4].

The use of patient-specific physical models in preoperative simulations allows surgeons to practice the procedure before entering the operating room, thereby boosting confidence and reducing intraoperative stress. These simulations also enable the testing of various surgical strategies to determine the most effective approach in terms of outcomes and scheduling. This process helps identify alternative surgical pathways and minimizes the risk of neurological deficits [5]. Various materials, including polyvinyl alcohol (PVA), polyhydroxybutyrat (PHY), agar, agarose, gelatin, alginate, and candle gel, have been explored for their suitability in simulating soft tissue. For instance, Forte et al. and Leibinger et al. developed a composite hydrogel to mimic brain tissue [6,7]. The selection of materials is determined by whether the goal is to replicate the brain’s elastic or viscoelastic properties, corresponding to the storage modulus and loss modulus, respectively. The storage modulus quantifies a material’s ability to store energy during deformation, reflecting the brain’s elastic behavior [8]. To replicate this characteristic, a composite of 6 wt.-% PVA and 1 wt.-% PHY1FT is optimal [9]. Conversely, the loss modulus measures the energy dissipated as heat during deformation, which is indicative of the brain’s viscoelastic properties [8]. For this, agarose (1 wt.-%) and GelMA (4 wt.-%) provide a more suitable approximation [9].

This study aims to develop a patient-specific, realistic simulation model for fluorescence-guided glioma resection, with a focus on adaptability to different tumor localizations. While the transsylvian resection of an insular glioma was used as an example, the model is designed to replicate various glioma resections by integrating fluorescence imaging to simulate 5-ALA-guided tumor visualization. After its development, the model was validated to assess user-friendliness and the effectiveness of skill transfer to real-life surgical scenarios. Subjective evaluations were conducted by neurosurgeons and senior physicians, while objective assessments of the materials were performed using rheological testing. Additionally, this study explored material science and sample preparation techniques to enhance the realism of brain tissue simulation.

## 2. Materials and Methods

This study protocol follows the SQUIRE 2.0 guidelines. Approval for the use of anonymized patient data was granted by the local ethics committee at the medical faculty of the Otto-von-Guericke University (Ethics approval number: NeuroCAM 146/19, date of approval 16 September 2019). ChatGPT-4 by OpenAI was used for language editing. As no identifiable information was used in this publication, further consent for participation and publication was not required. All data were gathered with patient consent as part of routine clinical care. The model is primarily constructed from a 3D-printed skull made using polylactic acid (PLA) material. Within this skull, brain tissue and glioma are integrated, with meticulous attention given to incorporating vascular structures. The subsequent sections will provide a detailed explanation of the precise manufacturing processes for each component. Based on extensive research [10,11,12], we selected agar-agar, candle gel, gelatin, and PVA in various concentrations as the materials for brain tissue simulation (Table 1).

### 2.1. Sample Preparation and Evaluation

#### 2.1.1. Hydrogel Preparation

##### PVA

To prepare the PVA hydrogel, PVA powder is mixed with distilled water at the desired concentration, as outlined in Table 1. The mixture is poured into a mold, refrigerated at 4 °C for eight hours, and then frozen at −20 °C for 24 h. The rheological properties of PVA are influenced by the number of freeze–thaw cycles applied [13].

##### Candle Gel

The candle gel is cut into small cubes for even melting, placed in a glass container, and melted in a microwave. Once fully melted, it is poured into a prepared mold.

##### Gelatin

To prepare the gelatin hydrogel, the desired amount of gelatin bloom 260 powder is measured, and cold water is added in the ratio specified in Table 1. The mixture is gently heated in a water bath while stirring until fully dissolved, then carefully poured into a mold.

##### Agar

Agar preparation involves mixing the appropriate amount of agar powder with water, based on the desired concentration in Table 1. The mixture is slowly heated in a water bath until fully dissolved, forming a clear solution, which is then carefully poured into a prepared mold.

#### 2.1.2. Subjective Evaluation of the Material Samples

**Figure 1:**
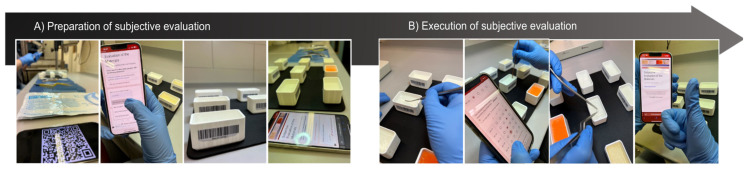
(**A**) Preparation for the subjective evaluation process, where two resident doctors and two senior physicians scanned QR codes to access a blinded Google Forms survey (**B**) Execution of the subjective evaluation, where two resident doctors and two senior physicians assessed the material samples based on criteria such as consistency, texture, elasticity, and resistance using microsurgical instruments.

Neurosurgeons evaluated the material samples based on several criteria. They scanned a QR code linked to a Google Forms survey, with the materials identified by a barcode system to keep the evaluators blinded (Figure 1).

Two resident doctors and two senior physicians assessed the samples for consistency, texture, elasticity, and resistance by palpating the materials with their hands and using microsurgical instruments. The evaluations were conducted on a 5-point Likert scale, with ‘Strongly Agree’ indicating the highest similarity to brain tissue. Survey data showed that candle gel, 12.5% gelatin, and 30% PVA most closely mimicked the haptic properties of brain tissue.

#### 2.1.3. Objective Evaluation of the Materials

**Figure 2:**
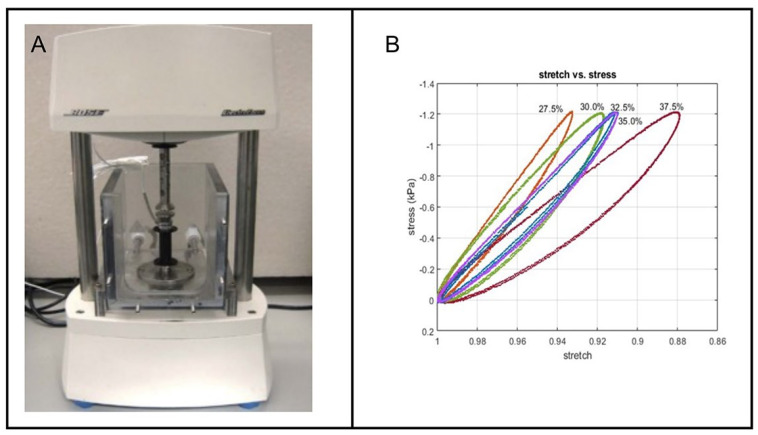
(**A**) The Bose ElectroForce System (New Castle, DE, USA) used for conducting three-cycle compression tests at a frequency of 0.1 Hz. (**B**) Stress vs. strain analysis of PVA at varying concentrations (27.5%, 30%, 32%, 35%, and 37.5%). These results were compared with previous measurements for candle gel and gelatin, revealing that candle gel exhibited higher stiffness, as indicated by its greater storage modulus.

We conducted rheological measurements of PVA at various concentrations and compared the results with previously recorded values for candle gel and gelatin. Five PVA concentrations were prepared, stored for one day, and subjected to a three-cycle compression test using the Bose ElectroForce System (New Castle, DE, USA) (Figure 2A) at a frequency of 0.1 Hz.

The stress–strain results for PVA were compared to those of candle gel and gelatin bloom 260 (Figure 2B). The data showed that candle gel had lower strain values, indicating a higher storage modulus and greater stiffness compared to the tested PVA and gelatin bloom 260 samples.

### 2.2. Construction of the Simulator and Simulation

**Figure 3:**
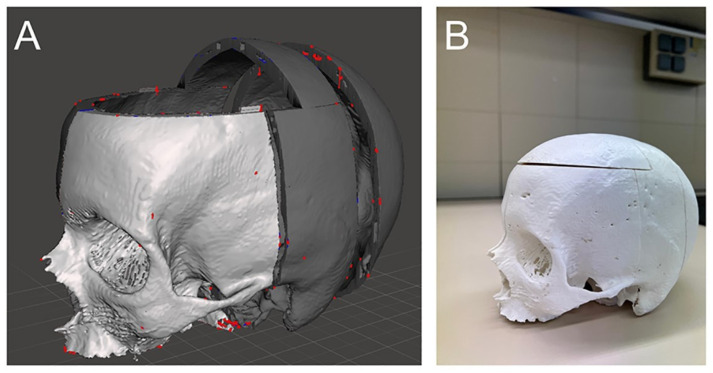
(**A**) Skull segmentation displayed in Meshmixer by Autodesk (San Francisco, CA, USA). (**B**) The complete 3D-printed skull following segmentation.

Patient-specific CT data in DICOM format were downloaded via Brainlab (Munich, Germany). The CT data were imported into InVesalius 3 by Centro de Tecnologia da Informação Renato Archer (Campinas, Brazil) for skull segmentation, then into Meshmixer, a 3D-freeware by Autodesk (San Francisco, CA, USA) (Figure 3A). The skull was divided into five parts: right parietal, left parietal, midsection, occipital, and roof sections. Connecting parts were created to join these sections (Figure 3B). The parts were 3D printed using a Raise3D Pro 2 printer by Raise3D (Costa Mesa, CA, USA) with 1.75 mm PLA filament.

**Figure 4:**
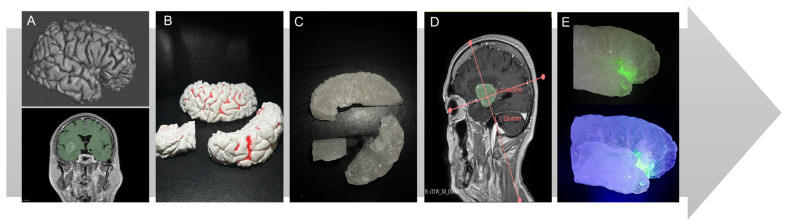
(**A**) The segmented right hemisphere in Meshmixer by Autodesk (San Francisco, CA, USA) and segmentation of the brain’s right hemisphere. (**B**) The 3D-printed sections of the Sylvian fissure. (**C**) Sylvian fissure sections cast in candle gel. (**D**) The insular glioma in MRI scan after Berger–Sanai. (**E**) The assembled Sylvian fissure sections with the integrated fluorescent glioma without blue light and with blue light.

After obtaining patient-specific MRI data from Brainlab Brainlab (Munich, Germany), we used 3D Slicer to segment the brain, focusing on the right hemisphere where the insular glioma was located, particularly the Sylvian fissure (Figure 4A) [14]. The right hemisphere was divided into three sections—frontal, temporal, and insular—using the “Create a Facegroup” function for precise modeling. The three resulting STL files were 3D-printed using the freeware Ideamaker and a Raise3D Pro2 printer by Raise3D (Costa Mesa, CA, USA) with a 1.75 mm PLA filament (Figure 4B). The printed models were secured in a container, and negative molds were created with WagnerSil 22 NF Premium Duplicating Silicone Rubber (Lonsee, Germany) to produce a reconstructed Sylvian fissure model using the described different materials (Figure 4C).

**Figure 5:**
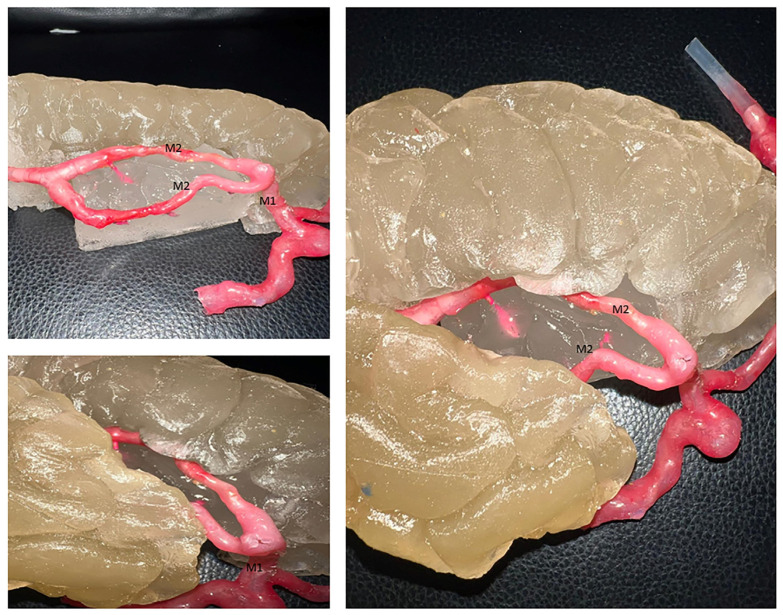
The relationships between the insula, the M1, M2 branches, and their perforators.

The chosen model represents an insular glioma classified according to the Berger-Sanai system, covering all four zones (I–IV) of the insula (Figure 4D). To create the glioma, we mixed candle gel hydrogel with 2% glycerin and poured it into the silicone mold. The addition of glycerine made the glioma slightly more elastic compared to brain tissue, as described by Bunevicius [15]. Fluorescent powder from Lumentics (Altenburg, Germany) was added to make the glioma glow green under blue light (Figure 4E). The cerebral vessels, made from silicone, were sourced from a separate project partially published by Amini et al. [16] and further elaborated upon in the work of Reiser et al. [17]. The relationships between the insula, the M2 branches, and their perforators are clearly illustrated in Figure 5.

**Figure 6:**
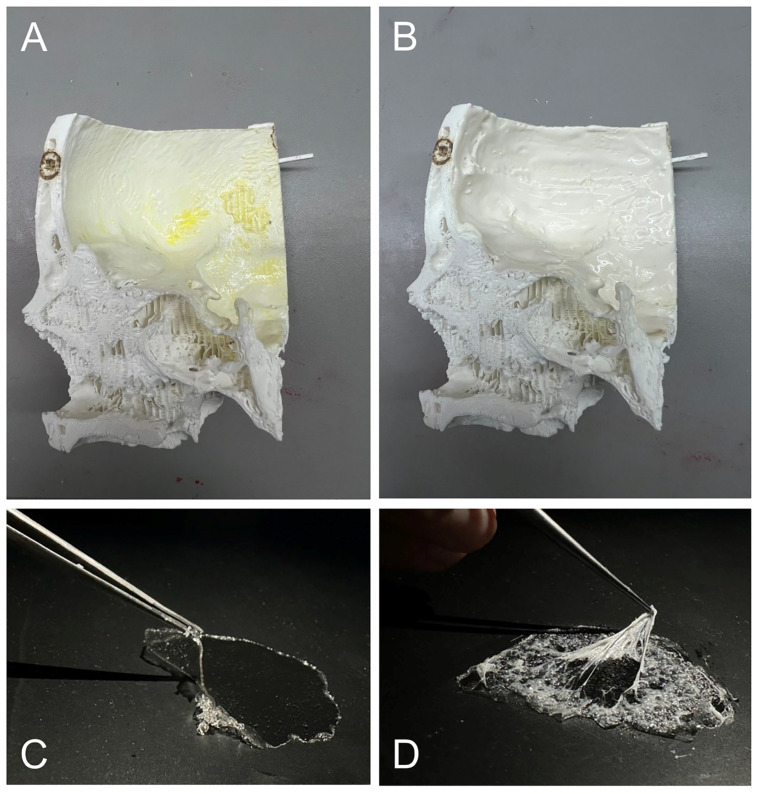
(**A**) The pre-treated inner surface of the skull with a latex coagulant. (**B**) Application of latex following the coagulant. (**C**) The first layer representing the pia mater. (**D**) The second layer representing the arachnoid mater.

After printing the individual skull parts, the interior of the pterional section was coated with Lilatex (Clausthal-Zellerfeld, Germany) thin liquid latex, also known as milk latex. The surface was pre-treated with a latex coagulant using a swab to ensure faster, more uniform latex application (Figure 6A). The latex was then carefully applied to the entire pterional section for consistent, realistic tactile feedback (Figure 6B).

The pia-arachnoid complex was recreated in two layers. The inner layer, representing the pia mater, was made from a high-concentration gelatin hydrogel (30 g gelatin with 100 mL water) (Figure 6C). The outer layer, simulating the arachnoid mater, was created by applying PVA hydrogel (20 g PVA with 200 mL water) over the first layer, followed by a spray adhesive application using UHU^®^ spray glue (Bühl, Germany) (Figure 6D).

#### 2.2.1. Simulation Process

Upon arrival at the microsurgical lab, participants received a brief overview of the simulation steps, with corresponding instruments introduced and detailed usage instructions provided. MRI images of the insular glioma were displayed and classified according to the Berger–Sanai system. Participants were then given 20 min to develop a surgical strategy and familiarize themselves with the instruments.

**Figure 7:**
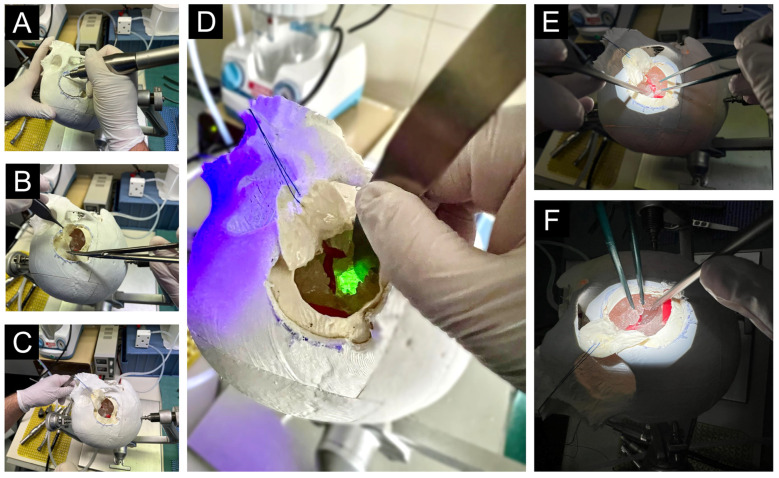
(**A**) Drilling the skull for craniotomy. (**B**) Circular opening of the dura mater. (**C**) Exposure of the surgical site and fixation of the opened dura with a clamp. (**D**) Exposure and resection of the fluorescent insular glioma. (**E**,**F**) Microsurgical dissection of the Sylvian fissure.

The skull model was then provided, and the simulation began with positioning the head in the Mayfield clamp by Integra LifeSciences Holdings Corporation (Princeton, NJ, USA). Participants outlined a pterional craniotomy on the skull and drilled a hole (Figure 7A). They used a dissector to separate the dura from the bone before completing the craniotomy and removing the clinoidal process with a bone rongeur.

Next, the dura was opened in a circular manner with scissors and forceps, secured with a stitch and clamp (Figure 7B,C). The microscope was adjusted, and the temporal lobe, frontal lobe, and Sylvian fissure were identified. A Sylvian vein could be dissected either from the frontal or temporal side. The Sylvian fissure was opened with bipolar forceps and a suction device (Figure 7E,F), after which the M2 branches were traced back to the middle cerebral artery (MCA). Finally, the resection of the insular glioma began under blue light, using bipolar forceps and a suction device for precise removal (Figure 7D).

#### 2.2.2. Subjective and Objective Evaluation

We evaluated the simulator’s face and content validity using a 5-point Likert scale questionnaire titled the “Insula Glioma Surgery Participant Survey”, focusing on overall usability and skill improvement.

**Figure 8:**
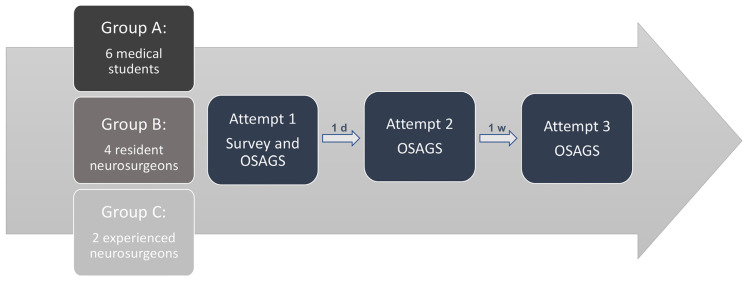
Evaluation process of the simulator using both subjective (Survey) and objective (OSAGS) tools. Participants were divided into three groups: Group A (medical students), Group B (resident neurosurgeons), and Group C (experienced neurosurgeons). Each participant completed three simulation attempts. The first attempt included a survey and OSAGS evaluation, followed by a second attempt after one day and a third attempt after one week, both assessed using the OSAGS tool.

Experienced neurosurgeons used the “Objective Structured Assessment of Glioma Surgery” (OSAGS), developed for this purpose and modeled after the mOSAACS score [18], to assess students during the trials. Each participant completed the simulation three times: the second attempt was one day after the first, and the third occurred one-week later. The OSAGS questionnaire was completed after the third attempt (Figure 8).

## 3. Results

### 3.1. Face and Content Validity

**Figure 9:**
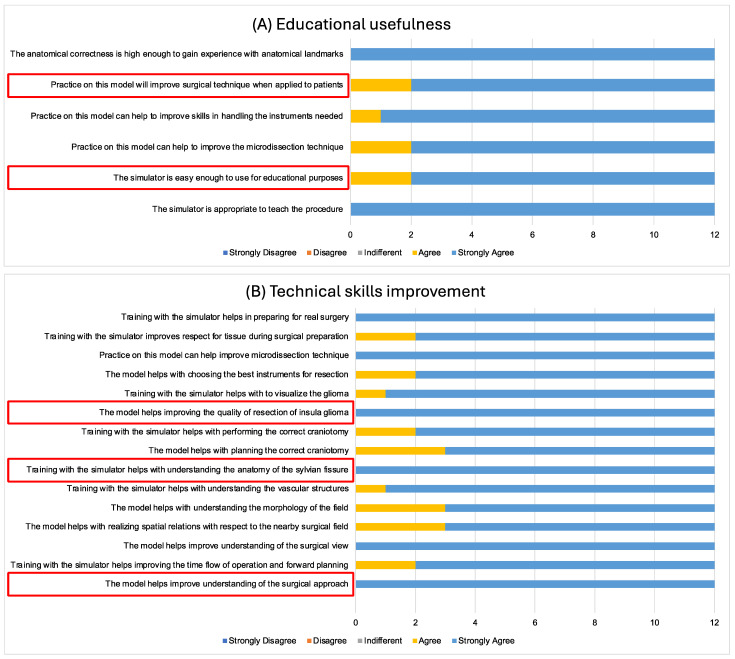
(**A**) Results from the “Educational usefulness” section of the survey. (**B**) Results from the “Technical skills improvement” section of the survey. The red frames highlight key aspects of the survey that demonstrate exceptionally strong results.

The “Educational Usefulness” section assessed the simulation’s effectiveness for learning. All twelve participants (100%) strongly agreed that the simulator is suitable for teaching the procedure, with twelve strongly agreeing and two agreeing that it is user-friendly. Ten participants strongly agreed and two agreed that it improves microdissection skills. For instrument handling, eleven strongly agreed and one agreed (Figure 9A).

The next section focused on “Perceived accuracy”, with questions primarily addressing realism and anatomical accuracy, allowing the simulator to be tested under realistic conditions. This section was only completed by Groups B and C, as they possess the necessary expertise to validate the simulator. For the statement, “The model replicates actual brain resection of glioma”, four participants (66.7%) strongly agreed, and two participants (33.3%) agreed. Regarding the statement, “The task is more difficult compared to real surgery”, 50% of participants were indifferent, while 50% disagreed. Conversely, for the statement, “The task is easier compared to real surgery”, 50% of participants were indifferent, and 50% agreed. A total of 83.3% of participants (5/6) strongly agreed with the statement, “Anatomical landmarks are easily identifiable”, while 16.6% (1/6) agreed. When asked if drilling into the 3D-printed skull felt realistic, five participants (83.3%) strongly agreed and one participant agreed. All participants strongly agreed with the statement, “The brain tissue feels realistic”. For the final statement in this section, “The tactile feedback of the brain while opening the Sylvian fissure feels realistic”, four participants strongly agreed and two agreed. The percentage values may appear less convincing due to the limited number of participants and the use of a 5-point Likert scale. However, it is important to note that no negative feedback was received regarding the realism of the model.

In the “Technical skills improvement” section, three specific statements were highlighted (marked in red in Figure 9B). All participants (100%) strongly agreed with each of these statements.

### 3.2. Construct Validity

**Figure 10:**
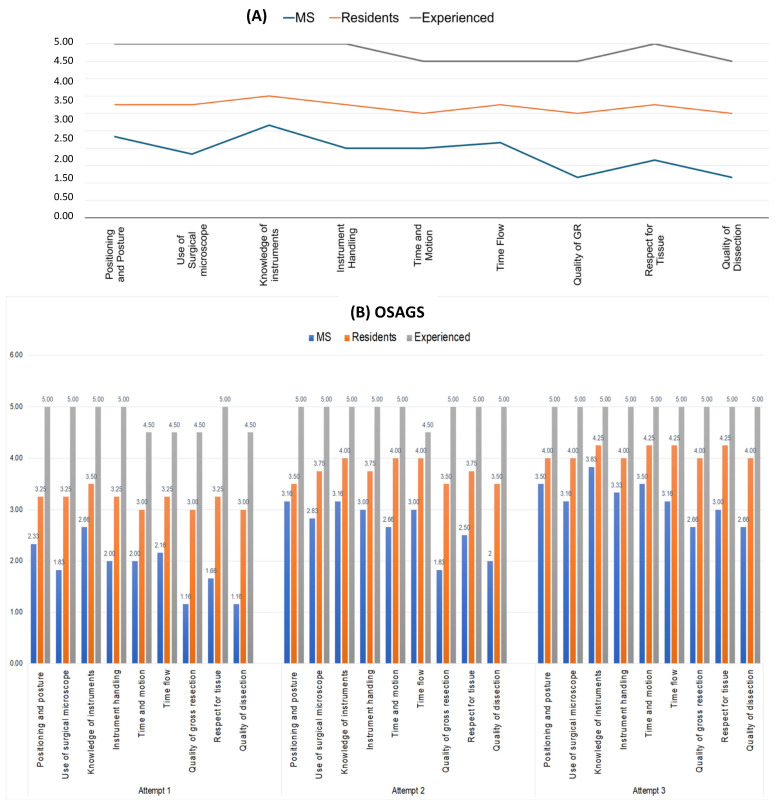
(**A**) OSAGS results from the first attempt, comparing medical students (blue), resident neurosurgeons (orange), and experienced neurosurgeons (grey). (**B**) OSAGS results across attempts 1, 2, and 3.

Figure 10A presents the mean scores of medical students (n = 6), resident neurosurgeons (n = 4), and experienced neurosurgeons (n = 2) during their first attempt at the objective assessments. As expected, the graph demonstrates high construct validity, accurately reflecting the varying experience levels among the groups.

The results indicate a noticeable acceleration in the learning curve, particularly among medical students and resident neurosurgeons, over the course of four attempts. Participants showed improvement with each successive attempt. For medical students, correlation analysis revealed statistically significant improvements between the first and second attempt (*p* = 0.045), the first and third attempt (*p* < 0.0001), and the second and third attempt (*p* = 0.013).

In contrast, the correlation analysis for resident neurosurgeons showed statistically significant improvements only between the first and third attempts (*p* < 0.001) and the second and third attempts (*p* = 0.031), but not between the first and second attempts (*p* = 0.728). Since the experienced neurosurgeons already scored highly on their first attempt, there was no significant improvement in subsequent attempts. Figure 10B provides an overview of the mean scores for all three groups across the three attempts.

### 3.3. Cost Analysis

The initial production of the model cost approximately EUR 139.30, mainly due to the high-quality silicone used for reusable negative molds. Subsequent simulations were significantly cheaper, at around EUR 4.50 each, as only a small skull section and a new insular glioma needed to be produced. Key equipment included a Raise3D Pro2 printer by Raise3D (Costa Mesa, CA, USA) (EUR 3500) and standard laboratory tools. Despite the upfront costs, the model’s reusability and low per-simulation expenses make it a cost-effective solution, especially for institutions with 3D printing facilities.

## 4. Discussion

Traditionally, surgical skills and manual dexterity have been developed in the operating room, directly on patients [19], which increases the risk of errors, especially for beginners. Novice surgeons are particularly prone to mistakes, while even experienced surgeons face challenges when testing new approaches on patients [20]. A 2015 study by Rolston and Bernstein reported that 14% of neurosurgical patients experience preventable perioperative complications [21]. Technical errors are linked to significant complications, higher morbidity, and increased economic costs [22]. To reduce these errors, simulators are essential, allowing surgeons to practice patient-specific procedures days before surgery. While cadaver simulations offer accurate anatomical models, they have limitations, including altered brain tissue texture, high costs, limited availability, and a lack of patient-specific pathologies [10,23,24].

While models simulating neurovascular and skull base surgeries are relatively common, 3D-printed constructs that replicate brain tumor resection and epilepsy surgery techniques are rarely documented. This suggests that 3D printing technology may still lack the capability to accurately replicate the intricate details required for these procedures [25]. Currently, 3D-printing in tumor surgery is mainly used for surgical planning, such as modeling diffuse low-grade gliomas and white matter tracts in relation to each other [26].

Only a few centers have undertaken the task of creating realistic phantom models for simulating neurosurgical tumor resection. For instance, Mashiko et al. used agar and gelatin to create a model capable of simulating tumors with different levels of hardness [27]. However, this study did not perform or construct face validity assessments, leaving the model unvalidated. Similarly, in a 2015 study, the same author developed a model to measure pressure during brain retraction but again did not validate the brain model, which could potentially lead to biased results [28].

The goal of this study was to develop a realistic simulation model to enhance participants’ technical skills in fluorescence-guided glioma resection. Fluorescence was used to replicate its critical role in real surgeries by enhancing tumor margin visualization, aiding in maximal safe resection, and improving surgical precision. It allows trainees to practice identifying tumor boundaries and simulates real intraoperative decision-making, thereby increasing the model’s realism and educational value. We observe the application of fluorescence in various training simulator models; however, these are often limited to simply created glioma models, typically integrated into cadaver models. This approach, while useful, limits innovation due to the constraints of cadaveric tissue, particularly regarding reusability, adaptability, and the realistic simulation of dynamic surgical scenarios [29,30,31]. By simulating an entire glioma operation, including the transsylvian resection of an insular glioma as an example, the model allows for the testing of alternative surgical approaches before application in real patients. The high face and content validity, assessed using a 5-point Likert scale, confirms that the simulator is both haptically and tactically applicable to real-world surgical scenarios. Furthermore, all participants agreed that this simulation model effectively supports the learning process. Even on the first attempt, the simulator demonstrated high construct validity based on the OSAGS, accurately reflecting participants’ skill levels. Over three attempts, a significant improvement in the learning curve was observed among medical students and neurosurgery residents, confirming that the simulator facilitates an effective learning process.

The phantom model material was evaluated both subjectively and objectively, resulting in a realistic and tactilely authentic representation of brain tissue. Particular emphasis was placed on modeling essential anatomical structures to enhance the training experience, including major vascular landmarks such as the MCA and the meninges. The Sylvian fissure was carefully reconstructed to allow for precise surgical practice, particularly in dissection techniques. The model’s reusability is ensured by the fact that the 3D-printed skull requires only a single production, with only the craniotomy section needing replacement for each simulation. The brain tissue remains consistent, and only the tumor is replaced at the same location for each participant (e.g., student or resident). This phantom model can be further expanded to incorporate additional structures and pathologies. Intra- and extra-axial brain tumors, as well as metastases, can be integrated based on their anatomical location, with the ability to include relevant vascular structures accordingly. Beyond glioma resection, the model offers potential applications in vascular simulations, such as aneurysm clipping, and in dural sinus reconstructions for meningioma surgery training. Future improvements will focus on enhancing the model’s usability by including instructional videos with step-by-step guidance, facilitating structured surgical training. Ongoing efforts also aim to systematically compare transsylvian and transcortical approaches, with plans for detailed video demonstrations in upcoming studies. While this study successfully validated the model’s development, its impact on real surgical performance remains to be evaluated. Future studies will assess its effectiveness in bridging the gap between simulation-based training and actual surgical procedures.

A limitation of this study is the use of a Likert scale for subjective evaluation. To enhance validation, we complemented this with objective rheological measurements. Future research will focus on developing standardized objective evaluation tools. Another limitation of this study is the use of non-perfused vessels in the current model. However, perfusion is technically feasible, as demonstrated in our previous aneurysm simulation by Julius et al. [17] An AVM model is also in development, featuring vessels that simulate bleeding upon rupture to enhance realism.

## 5. Conclusions

This study successfully developed a realistic, patient-specific simulator for fluorescence-guided glioma resection, with the flexibility to model tumors in various brain regions. By integrating fluorescence imaging, the simulator enhances the educational experience, allowing for improved visualization and resection techniques. It demonstrated strong face, content, and construct validity, significantly enhancing technical skills, particularly among medical students and junior residents. Its ability to replicate the tactile properties of brain tissue and key surgical landmarks makes it a valuable tool for neurosurgical training, providing a cost-effective and reusable alternative to traditional methods. Further integration of this model into neurosurgical curricula is warranted to refine complex surgical techniques and expand its application to different neurosurgical procedures.

## Figures and Tables

**Table 1:** Summary of tested materials, including the terminology used in the manuscript, brand names/sources, hydrogel concentrations, and preparation instructions for each concentration.

Material	Brand Name/Source	Concentrations of the Hydrogels (in %)
Polyvinyl alcohol–BF-04 (PVA)	COLLTEC© GmbH & Co. KG data (Bielefeld, Germany)	20
		22.5
		25
		27.5
		30
Gel wax/Candle gel 1 kg colorless (Candle gel)	KerzenKiste (Bullenkuhlen, Germany)	100
Gelatin Bloom 260 (Gelatin)	Local pig slaughterhouse	7.5
		12.5
		17.5
Agar-Agar (Agar)	Mr. P Ingredients (North Newbald, England)	2
		4
		6

## Data Availability

The data supporting the findings of this study are not openly available due to reasons of sensitivity and are available from the corresponding author upon reasonable request.

## Abbreviations

The following abbreviations are used in this manuscript:

5-ALA	5-aminolevulinic acid
PVA	polyvinyl alcohol
PHY	polyhydroxybutyrat
PLA	polylactic acid
MCA	middle cerebral artery
OSAGS	Objective Structured Assessment of Glioma Surgery

## References

[1] Przybylowski C.J., Hervey-Jumper S.L., Sanai N. (2021). Surgical Strategy for Insular Glioma. J. Neurooncol..

[2] Benet A., Hervey-Jumper S.L., Sánchez J.J.G., Lawton M.T., Berger M.S. (2016). Surgical Assessment of the Insula. Part 1: Surgical Anatomy and Morphometric Analysis of the Transsylvian and Transcortical Approaches to the Insula. J. Neurosurg..

[3] Safaee M.M., Englot D.J., Han S.J., Lawton M.T., Berger M.S. (2016). The Transsylvian Approach for Resection of Insular Gliomas: Technical Nuances of Splitting the Sylvian Fissure. J. Neurooncol..

[4] Barbosa B.J.A.P., Dimostheni A., Teixeira M.J., Tatagiba M., Lepski G. (2016). Insular Gliomas and the Role of Intraoperative Assistive Technologies: Results from a Volumetry-Based Retrospective Cohort. Clin. Neurol. Neurosurg..

[5] Mussi E., Mussa F., Santarelli C., Scagnet M., Uccheddu F., Furferi R., Volpe Y., Genitori L. (2020). Current Practice in Preoperative Virtual and Physical Simulation in Neurosurgery. Bioengineering.

[6] Forte A.E., Galvan S., Manieri F., Rodriguez Y Baena F., Dini D. (2016). A Composite Hydrogel for Brain Tissue Phantoms. Mater. Des..

[7] Leibinger A., Forte A.E., Tan Z., Oldfield M.J., Beyrau F., Dini D., Rodriguez Y Baena F. (2016). Erratum to: Soft Tissue Phantoms for Realistic Needle Insertion: A Comparative Study. Ann. Biomed. Eng..

[8] Li W., Shepherd D.E.T., Espino D.M. (2021). Investigation of the Compressive Viscoelastic Properties of Brain Tissue Under Time and Frequency Dependent Loading Conditions. Ann. Biomed. Eng..

[9] Tejo-Otero A., Fenollosa-Artés F., Achaerandio I., Rey-Vinolas S., Buj-Corral I., Mateos-Timoneda M.Á., Engel E. (2022). Soft-Tissue-Mimicking Using Hydrogels for the Development of Phantoms. Gels.

[10] Amini A., Zeller Y., Stein K.-P., Hartmann K., Wartmann T., Wex C., Mirzaee E., Swiatek V.M., Saalfeld S., Haghikia A. (2022). Overcoming Barriers in Neurosurgical Education: A Novel Approach to Practical Ventriculostomy Simulation. Oper. Neurosurg..

[11] Fromageau J., Gennisson J.-L., Schmitt C., Maurice R.L., Mongrain R., Cloutier G. (2007). Estimation of Polyvinyl Alcohol Cryogel Mechanical Properties with Four Ultrasound Elastography Methods and Comparison with Gold Standard Testings. IEEE Trans. Ultrason. Ferroelectr. Freq. Control..

[12] Madsen E.L., Hobson M.A., Shi H., Varghese T., Frank G.R. (2005). Tissue-Mimicking Agar/Gelatin Materials for Use in Heterogeneous Elastography Phantoms. Phys. Med. Biol..

[13] Taghizadeh S., Labuda C., Mobley J. (2018). Development of a Tissue-Mimicking Phantom of the Brain for Ultrasonic Studies. Ultrasound Med. Biol..

[14] Fedorov A., Beichel R., Kalpathy-Cramer J., Finet J., Fillion-Robin J.-C., Pujol S., Bauer C., Jennings D., Fennessy F.M., Sonka M. (2012). 3D Slicer as an Image Computing Platform for the Quantitative Imaging Network. Magn. Reson. Imaging..

[15] Bunevicius A., Schregel K., Sinkus R., Golby A., Patz S. (2020). REVIEW: MR Elastography of Brain Tumors. NeuroImage Clin..

[16] Amini A., Allgaier M., Saalfeld S., Stein K.-P., Rashidi A., Swiatek V.M., Sandalcioglu I.E., Neyazi B. (2024). Virtual Reality vs Phantom Model: Benefits and Drawbacks of Simulation Training in Neurosurgery. Oper. Neurosurg..

[17] Reiser J., Amini A., Swiatek V. (2025). How Good Is Neurosurgical Training?—Validation of a Perfused Microsurgical Aneurysm Training Simulator (MATS) Using a Modified OSAACS Score and Indocyanine-Green Angiography. Oper. Neurosurg..

[18] Belykh E., Miller E.J., Lei T., Chapple K., Byvaltsev V.A., Spetzler R.F., Nakaji P., Preul M.C. (2017). Face, Content, and Construct Validity of an Aneurysm Clipping Model Using Human Placenta. World Neurosurg..

[19] Cameron J.L. (1997). William Stewart Halsted. Our Surgical Heritage. Ann. Surg..

[20] Rolston J.D., Zygourakis C.C., Han S.J., Lau C.Y., Berger M.S., Parsa A.T. (2014). Medical Errors in Neurosurgery. Surg. Neurol. Int..

[21] Rolston J.D., Bernstein M. (2015). Errors in Neurosurgery. Neurosurg. Clin. N. Am..

[22] Stone S., Bernstein M. (2007). Prospective Error Recording in Surgery: An Analysis of 1108 Elective Neurosurgical Cases. Neurosurgery.

[23] Benet A., Plata-Bello J., Abla A.A., Acevedo-Bolton G., Saloner D., Lawton M.T. (2015). Implantation of 3D-Printed Patient-Specific Aneurysm Models into Cadaveric Specimens: A New Training Paradigm to Allow for Improvements in Cerebrovascular Surgery and Research. Biomed. Res. Int..

[24] Budday S., Sommer G., Haybaeck J., Steinmann P., Holzapfel G.A., Kuhl E. (2017). Rheological Characterization of Human Brain Tissue. Acta Biomater..

[25] Thiong’o G.M., Bernstein M., Drake J.M. (2021). 3D Printing in Neurosurgery Education: A Review. 3D Print. Med..

[26] Thawani J.P., Singh N., Pisapia J.M., Abdullah K.G., Parker D., Pukenas B.A., Zager E.L., Verma R., Brem S. (2017). Three-Dimensional Printed Modeling of Diffuse Low-Grade Gliomas and Associated White Matter Tract Anatomy. Neurosurgery.

[27] Mashiko T., Oguma H., Konno T., Gomi A., Yamaguchi T., Nagayama R., Sato M., Iwase R., Kawai K. (2018). Training of Intra-Axial Brain Tumor Resection Using a Self-Made Simple Device with Agar and Gelatin. World Neurosurg..

[28] Mashiko T., Konno T., Kaneko N., Watanabe E. (2015). Training in Brain Retraction Using a Self-Made Three-Dimensional Model. World Neurosurg..

[29] Regelsberger J., Eicker S., Siasios I., Hänggi D., Kirsch M., Horn P., Winkler P., Signoretti S., Fountas K., Dufour H. (2015). In Vivo Porcine Training Model for Cranial Neurosurgery. Neurosurg. Rev..

[30] Valli D., Belykh E., Zhao X., Gandhi S., Cavallo C., Martirosyan N.L., Nakaji P., Lawton M.T., Preul M.C. (2019). Development of a Simulation Model for Fluorescence-Guided Brain Tumor Surgery. Front. Oncol..

[31] Kamp M.A., Knipps J., Steiger H.-J., Rapp M., Cornelius J.F., Folke-Sabel S., Sabel M. (2015). Training for Brain Tumour Resection: A Realistic Model with Easy Accessibility. Acta Neurochir..

